# The* In Vitro* Antimicrobial Effects of* Lavandula angustifolia* Essential Oil in Combination with Conventional Antimicrobial Agents

**DOI:** 10.1155/2016/2752739

**Published:** 2016-11-06

**Authors:** Stephanie de Rapper, Alvaro Viljoen, Sandy van Vuuren

**Affiliations:** ^1^Department of Pharmacy and Pharmacology, Faculty of Health Sciences, University of the Witwatersrand, 7 York Road, Parktown 2193, South Africa; ^2^Department of Pharmaceutical Sciences, Faculty of Science, Tshwane University of Technology, Pretoria, South Africa; ^3^SAMRC Herbal Drugs Research Unit, Faculty of Science, Tshwane University of Technology, Pretoria, South Africa

## Abstract

The paper focuses on the* in vitro* antimicrobial activity of* Lavandula angustifolia *Mill. (lavender) essential oil in combination with four commercial antimicrobial agents. Stock solutions of chloramphenicol, ciprofloxacin, nystatin, and fusidic acid were tested in combination with* L. angustifolia *essential oil. The antimicrobial activities of the combinations were investigated against the Gram-positive bacterial strain* Staphylococcus aureus* (ATCC 6538) and Gram-negative* Pseudomonas aeruginosa* (ATCC 27858) and* Candida albicans* (ATCC 10231) was selected to represent the yeasts. The antimicrobial effect was performed using the minimum inhibitory concentration (MIC) microdilution assay. Isobolograms were constructed for varying ratios. The most prominent interaction was noted when* L. angustifolia* essential oil was combined with chloramphenicol and tested against the pathogen* P. aeruginosa *(ΣFIC of 0.29).* Lavendula angustifolia *essential oil was shown in most cases to interact synergistically with conventional antimicrobials when combined in ratios where higher volumes of* L. angustifolia *essential oil were incorporated into the combination.

## 1. Introduction

The art of using essential oils for therapeutic practice has an extensive history reaching many continents around the world [1]. The practice of aromatherapy and the use of essential oils for therapeutic purposes has maintained its popularity through the ages and continued today with essential oils used as a popular alternate therapy for antimicrobial effects. The increased interest in aromatherapy for these purposes has launched essential oils into the international markets, where they are often sold as “natural antibiotics” [2, 3].

*Lavendula angustifolia*, considered the most versatile and popular of all essential oils used in aromatherapy, is well documented [4–8]. Within the field of aromatherapy,* L. angustifolia* essential oil is believed to have antimicrobial value for a plethora of conditions ranging from skin inflictions such as boils and yeast infections to respiratory complaints such as bronchitis and whooping cough [9–11]. The therapeutic use of* L. angustifolia *essential oil could be traced back to as early as Roman and Greek times, where it was frequently used medicinally due to the apparent lack of toxicity [11].* Lavandula *species have also been proven in literature to have the potential to treat bacterial infections commonly associated with resistance to conventional antimicrobial agents. At a concentration of less than 1%,* L. angustifolia *essential oil inhibits the growth of microorganisms such as methicillin-resistant* Staphylococcus aureus *and vancomycin-resistant* Enterococcus faecalis* [12].

There have been a number of studies on the antimicrobial activities of essential oils in combination with conventional antibacterial agents [13–20]. A study conducted in 2014 aimed to identify the antimicrobial properties of 35 essential oil-antimicrobial combinations. The study investigated the effects of some popular essential oils such as lavender (*Lavandula angustifolia*) and tea-tree (*Melaleuca alternifolia* (Maiden & Betche) Cheel.), and less popular oils such as cinnamon bark (*Cinnamomum zeylanicum* Blume) and marjoram (*Origanum majorana *L.) [21]. These oils were tested in combination with common *β*-lactam penicillin antibiotics and the structurally similar cephalosporins. Of the 35 combinations tested, only four were identified as synergistic.* L. angustifolia* essential oil in combination with piperacillin against* Escherichia coli* showed the greatest level of synergy with an ΣFIC value of 0.26.

Despite the popularity and proven activity of* L. angustifolia* essential oil, no studies could be found that have focused on the antimicrobial potential of* L. angustifolia* essential oil in combination with non-beta lactam antimicrobial agents. With this in mind, the essential oil of* L. angustifolia* was investigated in combination with four commercially available antimicrobial agents (nystatin, chloramphenicol, ciprofloxacin, and fusidic acid) to determine what potential interaction may occur should they be used in combination.

## 2. Materials and Methods 

The composition of* L. angustifolia* Mill. (Robertet®) was confirmed using gas chromatography coupled to a mass spectrometer and flame ionization detector (GC-MS-FID). The GCMS-FID (Agilent 6890N GC system and 5973 MS) was equipped with a HP-Innowax polyethylene glycol column (60 m × 250 *μ*m i.d. × 0.25 *μ*m film thickness). A volume of 1 *μ*L of the essential oil was injected (using a split ratio of 200 : 1) with an autosampler at 24.79 psi and an inlet temperature of 250°C. The GC oven temperature was set at 60°C for 10 minutes and then 220°C at a rate of 4°C/minute for 10 minutes and followed by a temperature of 240°C at a rate of 1°C/minute. Helium was used as a carrier gas at a constant flow of 1.2 mL/minute. Spectra were obtained on electron impact at 70 eV, scanning from 35 to 550 *m*/*z*. The percentage composition of the individual components was quantified by integration measurements, using flame ionization detection (FID, 250°C). Component identifications were made by comparing mass spectra from the total ion chromatogram and retention indices using NIST and Mass Finder GC-MS libraries.

For the antimicrobial analysis of* L. angustifolia* essential oil in combination with conventional antimicrobial agents, stock solutions of ciprofloxacin (≥98.0% purity, Sigma-Aldrich), nystatin (70.0% purity, Sigma-Aldrich), and fusidic acid (≥98.0% purity, Sigma-Aldrich) were made up to a concentration of 0.01 mg/mL using sterile water as the diluent. A 70% ethanol solution was used initially as a solvent for chloramphenicol (≥98.0% purity, Sigma-Aldrich) and thereafter diluted in sterile water. These antimicrobial agents were selected for analysis due to their indication in respiratory and topical infections. Chloramphenicol was selected due to its indication in the treatment of serious Gram-negative bacterial infections [22]. Chloramphenicol was also investigated against* C. albicans* as it has been indicated in previous literature as having substantial antifungal activity [23]. Ciprofloxacin, a first-generation fluoroquinolone antibiotic, was selected due to its broad-spectrum activity. Fusidic acid was selected due to its specific use in the treatment of Gram-positive bacterial infections, while nystatin was chosen to represent the class of antifungal agents [22]. The oil of* L. angustifolia* was prepared to yield a stock concentration of 32 mg/mL in acetone. The following ratios (in *μ*L) of* L. angustifolia* essential oil and the antimicrobial agents under analysis were investigated (*L. angustifolia* essential oil: antimicrobial agent): 90 : 10, 80 : 20, 70 : 30, 60 : 40, 50 : 50, 40 : 60, 30 : 70, 20 : 80, and 10 : 90. For each ratio tested, the essential oil and antimicrobial concentration varied and the exact concentration for each ratio is given in Table 1. The antimicrobial activities of the combinations were investigated against the Gram-positive bacterial strain* Staphylococcus aureus* (ATCC 6538) and Gram-negative strain* Pseudomonas aeruginosa* (ATCC 27858) and* Candida albicans* (ATCC 10231) was selected to represent the yeasts. The antimicrobial effect was determined by means of the minimum inhibitory concentration (MIC) microdilution assay [24]. The microtitre plates were prepared by adding 100 *μ*L of sterile, distilled water into each of the wells. The antimicrobial agents and* L. angustifolia* essential oil were added to the first row, at a volume of 100 *μ*L (where they were tested individually) and 50 : 50 *μ*L (where they were tested in equal volume combinations). Negative controls (water/acetone) were included in each assay to determine the antimicrobial activity of the solvent. Sterile media, such as Tryptone Soya Broth (TSB), and culture controls were included to confirm sterility and viability, respectively. The Clinical and Laboratory Standards Institute (CLSI) [25] and KnowledgeBase [26] were used to compare antimicrobial break points of the MIC values obtained from antimicrobial testing. This is undertaken to ensure that the pathogen responded to the tested antimicrobial in a reproducible manner and that the MIC values were within the recommended ranges (Table 3). Cultures were added to all the wells of their respective microtitre plates, at a concentration of 1 × 10^6^ colony forming units (CFU)/mL. The microtitre plates were incubated under optimal conditions of 37°C for 24 hours for bacteria and 37°C for 48 hours for the yeast. After incubation,* p-*iodonitrotetrazolium violet solution (INT) at a concentration of 0.4 mg/mL was added into each well (40 *μ*L), upon which colour changes were noted approximately six hours later for the bacteria and 24 hours later for the yeast. The lowest concentration identified with no growth or colour change was determined as the MIC. The fractional inhibitory concentration (FIC) and the FIC index (ΣFIC) were calculated to determine the interaction between* L. angustifolia* essential oil and the selected antimicrobial agents. The ΣFIC for each combination was interpreted as synergistic where ΣFIC was less than or equal to 0.50. For additive properties, ΣFIC was interpreted as greater than 0.50 but less than or equal to 1.00. For indifference, ΣFIC values are greater than 1.00 but less than or equal to 4.00 and antagonism occurs when ΣFIC greater than 4.00 is observed [27].

For the varied concentrations of* L. angustifolia* essential oil, antibiotic combination, isobolograms were constructed using GraphPad Prism, version five® software to present the mean MIC values of the combinations as ratios [28]. The isobolograms were interpreted by examining the data points for each ratio in relation to the MIC values for the oils independently. All points between the 0.5 : 0.5 and 1.0 : 1.0 line were interpreted as additive and above the 1.0 : 1.0 line and below the 4.0 : 4.0 line were classified as noninteractive. The points below or on the 0.5 : 0.5 line on the isobologram were interpreted as synergistic with antagonism noted for data points above the 4.0 : 4.0 line [27]. All tests were undertaken in duplicate and when results differed by more than one dilution factor, a third replicate was undertaken.

## 3. Results and Discussion

The chemical composition of* L. angustifolia* essential oil was determined to confirm the specific chemotype (Table 2). A full chemical profile was established for this essential oil, with the major constituents, linalyl acetate (36.7%), linalool (31.4%), and terpinen-4-ol (14.9%), identified. The chemical composition of* L. angustifolia* essential oil has been studied extensively in literature and the composition reported here is congruent with literature [29–32].

Initially, confirmation of the antimicrobial activity of the four antimicrobial agents was undertaken against the three test microorganisms (Table 3). Where possible, the MIC values determined were congruent with breakpoint expectations. Only* S. aureus* demonstrated a marginally higher susceptibility pattern (0.11 *μ*g/mL) than the expected range (0.12–0.50 *μ*g/mL) for ciprofloxacin, but given that the assay is working with doubling dilutions, this is not significant.

The mean MIC values obtained for* L. angustifolia* essential oil against the tested pathogens were 3.00 mg/mL, 2.00 mg/mL, and 2.00 mg/mL for* C. albicans*,* S. aureus, *and* P. aeruginosa,* respectively. Synergy was noted for two interactions,* L. angustifolia* essential oil in combination with ciprofloxacin against* S. aureus* (ΣFIC of 0.49) and* L. angustifolia* essential oil in combination with chloramphenicol against* P. aeruginosa* (ΣFIC of 0.29) (Table 4). Before this study, no research has been conducted on the antimicrobial interaction between* L. angustifolia* essential oil and chloramphenicol, nor chloramphenicol in combination with other natural therapies against* P. aeruginosa.* Chloramphenicol has, however, been placed in combination with other essential oils and tested against other microorganisms. A study conducted in 2013 determined the antimicrobial effect of chloramphenicol in combination with* Coriandrum sativum *L. essential oil against* Acinetobacter baumannii* using the ΣFIC analysis [33]. This combination also demonstrated considerable synergistic antimicrobial potential with a ΣFIC value of 0.28. The antimicrobial potential of this agent in combination with essential oil compounds is further augmented by a study conducted by Halawani [34]. According to Halawani [34], chloramphenicol in combination with thymoquinone and thymohydroquinone (the major chemical constituents of the essential oil* Nigella sativa *L.) demonstrated synergistic antimicrobial effects against* E. coli*,* S. aureus*,* P. aeruginosa,* and* Salmonella typhimurium.* These studies further demonstrate the antimicrobial potential of this antimicrobial agent in combination with essential oils.

**Figure 1:**
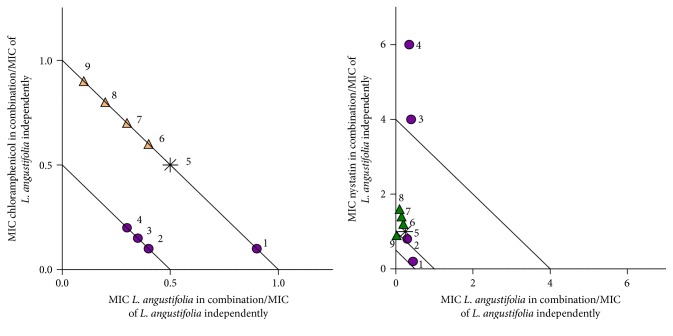
Isobologram representation of* L. angustifolia* essential oil in combination with chloramphenicol and nystatin at various ratios against* C. albicans* (ATCC 10231). ● indicates* L. angustifolia* essential oil in majority volume, ▲ indicates the antimicrobial agent in majority volume, and *∗* indicates equal volume of* L. angustifolia* essential oil to antimicrobial agent. Points 1–9 (Table 1) provide exact concentrations for antimicrobial and lavender oil.

**Figure 2 fig2:**
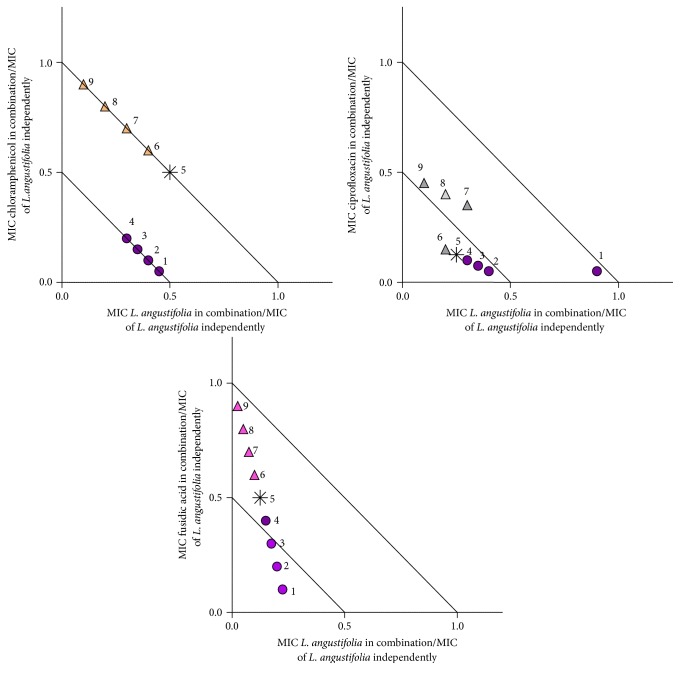
Isobologram representation of* L. angustifolia* essential oil in combination with chloramphenicol, ciprofloxacin, and fusidic acid at various ratios against* S. aureus* (ATCC 6538). ● indicates* L. angustifolia* essential oil in majority volume, ▲ indicates the antimicrobial agent in majority volume, and *∗* indicates equal volume of* L. angustifolia* essential oil to antimicrobial agent. Points 1–9 (Table 1) provide exact concentrations for antimicrobial and lavender oil.

**Figure 3:**
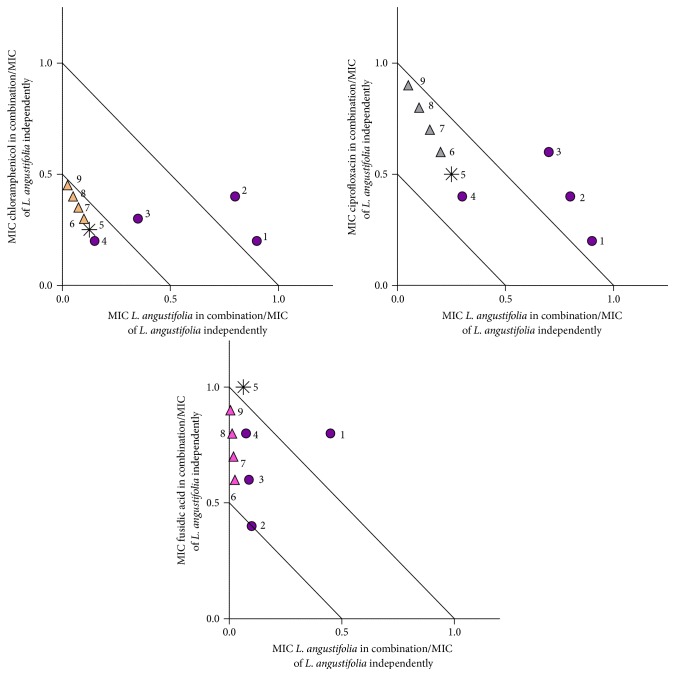
Isobologram representation of* L. angustifolia* essential oil in combination with chloramphenicol, ciprofloxacin, and fusidic acid at various ratios against* P. aeruginosa* (ATCC 27858). ● indicates* L. angustifolia* essential oil in majority volume, ▲ indicates the antimicrobial agent in majority volume, and *∗* indicates equal volume of* L. angustifolia* essential oil to antimicrobial agent. Points 1–9 (Table 1) provide exact concentrations for antimicrobial and lavender oil.

In order to determine what interactions could be apparent when the concentration of oil and conventional antibiotic varies, further investigation was undertaken and plotted on isobolograms (Table 1, Figures 1 [Fig fig2]–3). When investigating the combination of chloramphenicol and nystatin with* L. angustifolia* essential oil (Figure 1), synergy was noted for three of the nine ratios with chloramphenicol :* L. angustifolia* essential oil (20 : 80, 30 : 70, and 40 : 60) and one synergistic interaction (20 : 80) for nystatin :* L. angustifolia* essential oil. For the combination with chloramphenicol, synergy was more apparent where* L. angustifolia* essential oil was present in higher volume ratios. Indifference was mostly observed for the combinations of nystatin with* L. angustifolia* essential oil. Two ratios demonstrated antagonism (where nystatin is combined with* L. angustifolia* essential oil) at ratios of 20 : 80 and 30 : 70.

Against* S. aureus*,* L. angustifolia* essential oil was combined with the antimicrobial agents chloramphenicol, ciprofloxacin, and fusidic acid (Figure 2). Synergy was observed for the combination of chloramphenicol and* L. angustifolia* essential oil in four of the ratios investigated (chloramphenicol :* L. angustifolia* essential oil 10 : 90, 20 : 80, 30 : 70, and 40 : 60), with a predominantly additive relationship identified for the remainder of the other combinations tested. When placed in combination with ciprofloxacin at varying ratios, five of the nine ratios investigated were identified as synergistic (40 : 60, 50 : 50, 60 : 40, 70 : 30, and 20 : 80). The combination of fusidic acid and* L. angustifolia* essential oil varied between additive and synergistic, depending on the ratio. Synergy was observed for three of the ratios investigated (10 : 90, 20 : 80, and 30 : 70).

When investigated against* P. aeruginosa*, the* L. angustifolia* essential oil combinations with chloramphenicol, ciprofloxacin, and fusidic acid demonstrated varied interactions (Figure 3). Mainly synergistic antimicrobial interactions occurred with six of the nine ratios investigated for the combination of* L. angustifolia* essential oil with chloramphenicol. When placed in combination with ciprofloxacin, additive effects were predominant. Similarly, the combination of fusidic acid and* L. angustifolia* essential oil in various combinations demonstrated mainly additive interactions, with synergy identified for one ratio (20 : 80).

Potentiation of an antimicrobial agent results when a technique is applied to the antimicrobial agent causing it to demonstrate a greater antimicrobial effect at a lower concentration [35]. A number of studies have been conducted on the potential of plants to potentiate antimicrobial agents [36–45]. One study determined that* Rosmarinus officinalis *L. essential oil potentiates the antimicrobial activity of erythromycin 16–32-fold against* S. aureus* [40].

The most promising combination is noted for* L. angustifolia* essential oil with fusidic acid against* S. aureus* in which the antimicrobial effect of this agent is potentiated 5-fold. Fusidic acid is primarily applied in the treatment of topical* Staphylococcal* infections; therefore the potentiation of this antimicrobial agent suggests promise for the use of this combination within future pharmaceutical preparations.

When combined with chloramphenicol, it was noted that ratios higher in* L. angustifolia* essential oil were responsible for the synergistic antimicrobial effects of the combination; however, an antagonistic interaction was observed with the combination of nystatin and* L. angustifolia* essential oil. This demonstrates that, in spite of the positive results obtained against some pathogens, caution should still be exercised. The safety of combining complementary therapy and conventional prescription medications has been of concern [46]. A study conducted by Van Vuuren et al. [47] substantiates this point as the combination of* M. alternifolia* essential oil and ciprofloxacin against the microorganism* S. aureus* was shown to yield an antagonistic antimicrobial effect with ΣFIC values ranging from 5.17 to 7.70, dependant on the concentration at which these agents were combined.

Ciprofloxacin is indicated in the treatment of many bacterial infections including* S. aureus* related infections [22], while* L. angustifolia* essential oil is indicated mainly for the treatment of superficial skin infections, many of which originate from a* Staphylococcal* source [9–11]. Interestingly, this combination showed that* L. angustifolia* essential oil potentiated the antimicrobial effects of ciprofloxacin suggesting some therapeutic benefit in combining these agents. When investigated against* P. aeruginosa*,* L. angustifolia* essential oil was placed in combination with chloramphenicol, ciprofloxacin, and fusidic acid.

The outcomes obtained indicate that* L. angustifolia* essential oil has the potential to improve the antibacterial effects of some commercial antibiotics. This may be as a result of the interactions occurring between the chemical compounds of* L. angustifolia* essential oil and the antimicrobial agents used. Due to the complexity of essential oil chemistry it has been determined that essential oils have the ability to affect any number of antimicrobial pathways. Studies on the effects of the major chemical constituents of* L. angustifolia* essential oil, linalool, linalyl acetate, and terpinen-4-ol, indicate the mechanism of action of these components to comprise mainly of damage to the lipid layer of the cell membrane, resulting in bacterial cell leakage [48–50]. Although some individual chemical compounds have been determined as having antimicrobial potential independently, the effects of the essential oils are predominantly due to the interactions of the chemical entities [51]. The antimicrobial agent most potentiated by* L. angustifolia* essential oil was fusidic acid. Fusidic acid causes damage to bacterial cells by reducing protein synthesis [22]. The combination of* L. angustifolia* essential oil and fusidic acid highlights the potential of multitarget effects between essential oils and their chemistry when used in combination with conventional antimicrobial agents. In order for antimicrobial resistance to be overcome, such a mechanism is required in proposed combinations in order to render a greater antimicrobial effect [52]. This interaction is further observed amongst the combinations eliciting additive and synergistic effects such as* L. angustifolia* essential oil in combination with ciprofloxacin (inhibiting DNA enzymes [22]) and* L. angustifolia* essential oil in combination with chloramphenicol (inhibiting protein synthesis [22]). One needs to remain cognisant that synergistic interactions may not necessarily be as a result of the major compounds (only) but minor compounds may also play a role in the positive interactions observed.

## 4. Conclusion

Antimicrobial resistance has been on the rise over recent years with an unknown number of resistant bacterial strains developed. The microorganism* Enterococcus faecium*, responsible for causing infections such as endocarditis, cellulitis, and bacteremia [22], had, as of 2009, developed a high level of resistance to aminoglycoside antibiotics. No known alternative for treatment currently exists [53]. Due to this growing level of resistance, research has been undertaken to develop new products with antimicrobial potential for use independently and in combination with current antibiotics, with researchers looking towards natural products and plants for solutions [54]. The lack of antagonism and indication of possible drug potentiation within this study further augments the potential for the use of essential oils in combination with antimicrobial agents for a synergistic antimicrobial effect. This study suggests some therapeutic potential in combining* L. angustifolia* essential oil with common antimicrobial agents in the future.

## Figures and Tables

**Table 1:** The concentrations of essential oil and antimicrobial agent associated with the volume ratios studied.

Plot number^*∗*^	Volume ratio of essential oil : antimicrobial agent	Concentration of antimicrobial agent in combination	Concentration of essential oil in combination
	*μ*L	*μ*g/mL	mg/mL
1	90 : 10	0.25	7.2
2	80 : 20	0.50	6.4
3	70 : 30	0.75	5.6
4	60 : 40	1.00	4.8
5	50 : 50	1.25	4.0
6	40 : 60	1.50	3.2
7	30 : 70	1.75	2.4
8	20 : 80	2.00	1.6
9	10 : 90	2.25	0.8

^*∗*^Referring to points on isobologram graphs.

**Table 2:** The chemical composition of *Lavandula angustifolia *Mill. essential oil.

RRI	Constituents	Percentage abundance
1016	*α-*Pinene	0.1
1019	*α-*Thujene	t.a.^*∗*^
1057	Camphene	0.1
1104	*β-*Pinene	t.a.^*∗*^
1159	Myrcene	0.2
1193	*α-*Terpinene	t.a.^*∗*^
1194	Limonene	0.1
1202	Eucalyptol	0.5
1232	*β*-*trans*-Ocimene	3.0
1242	*γ-*Terpinene	0.1
1250	*β*-*cis*-Ocimene	2.1
1319	3-Octanone	0.4
1270	*p-*Cymene	0.1
1281	Terpinolene	t.a.^*∗*^
1331	Hexyl butyrate	0.1
1372	Allo-ocimene	0.5
1376	1-Octenyl acetate	0.8
1385	3-Octanol	0.1
1411	*n-*Hexyl butyrate	0.3
1441	Hexyl-2-methylbutyrate	t.a.^*∗*^
1447	*cis-*Linalool oxide	0.2
1471	*cis-*Linalool oxide	0.1
1521	Camphor	0.3
1541	Linalool	31.4^†^
1563	Linalyl acetate	36.7^†^
1572	*α-*Cedrene	t.a.^*∗*^
1573	*α-*Santalene	0.4
1584	*α-*Bergamotene	0.3
1602	Terpinen-4-ol	14.9^†^
1665	*β-*Farnesene	1.4
1677	Lavandulol	1.2
1680	Cryptone	0.2
1701	*α-*Terpineol	0.3
1702	Borneol	0.7
1741	Carvone	t.a.^*∗*^
1763	*γ-*Cadinene	0.2
1797	Cuminaldehyde	0.1
1855	*p-*Cymen-8-ol	0.3
2010	Caryophyllene epoxide	t.a.^*∗*^
2225	Thymol	0.1

	Total	97.30%

^*∗*^t.a. indicates trace amounts; † indicates major chemical constituents.

**Table 3:** Mean MIC values in *μ*g/mL (*n* = 3) for the antimicrobial agents selected for combination analysis.

Antimicrobial agent	*C. albicans*		*S. aureus*		*P. aeruginosa*	
	ATCC 10231		ATCC 6538		ATCC 27858	
	Value obtained	Break point range^*∗*^	Value obtained	Break point range	Value obtained	Break point range
Chloramphenicol	0.63	<4.00	0.31	0.10–15.60	0.31	0.06–32.00
Ciprofloxacin	NA^†^	NA	0.11	0.12–0.50	0.04	<1.00–2.50
Fusidic acid	NA	NA	0.63	0.10–>32.00	0.31	≤1.00
Nystatin	0.16	≤4.00	NA	NA	NA	NA

^*∗*^Break point ranges for commercial antimicrobials as determined by the KnowledgeBase antimicrobial index, CLSI guidelines, and EUCAST antimicrobial index; ^†^NA: not applicable as microorganisms are not susceptible to these antimicrobial agents.

**Table 4:** The antimicrobial effect of the individual components within the essential oil : antimicrobial combination.

Microorganism	MIC individual			ΣFIC	Interpretation
	LA	Antimicrobial			
	(mg/mL)	(*μ*g/mL)			
*C. albicans* (ATCC 10231)	3.00	CH	0.63	1.00	Additive
		N	0.16	1.14	Noninteractive

*S. aureus* (ATCC 6538)	2.00	CH	0.31	0.75	Additive
		C	0.11	0.49	Synergistic
		FA	0.63	0.65	Additive

*P. aeruginosa* (ATCC 27858)	2.00	CH	0.31	0.29	Synergistic
		C	0.04	0.74	Additive
		FA	0.31	1.13	Noninteractive

LA indicates *L. angustifolia *essential oil, CH indicates chloramphenicol, N indicates nystatin, C indicates ciprofloxacin, and FA indicates fusidic acid.
